# Development of an imaged capillary isoelectric focusing method for characterizing the surface charge of mRNA lipid nanoparticle vaccines

**DOI:** 10.1002/elps.201900063

**Published:** 2019-07-24

**Authors:** John W. Loughney, Kevin Minsker, Sha Ha, Richard R. Rustandi

**Affiliations:** ^1^ Vaccine Analytical Research & Development Merck and Co. Inc. West Point PA USA

**Keywords:** Cationic lipid, Imaged capillary isoelectric focusing, Isoelectric point (pI), Lipid nanoparticles, Maurice, mRNA vaccine

## Abstract

Lipid nanoparticles (LNPs) have been employed for drug delivery in small molecules, siRNA, mRNA, and pDNA for both therapeutics and vaccines. Characterization of LNPs is challenging because they are heterogeneous mixtures of large complex particles. Many tools for particle size characterization, such as dynamic and static light scattering, have been applied as well as morphology analysis using electron microscopy. CE has been applied for the characterization of many different large particles such as liposomes, polymer, and viruses. However, there have been limited efforts to characterize the surface charge of LNPs and CIEF has not been explored for this type of particle. Typically, LNPs for delivery of oligonucleotides contain at least four different lipids, with at least one being an ionizable cationic lipid. Here, we describe the development of an imaged capillary isoelectric focusing method used to measure the surface charge (i.e., pI) of an LNP‐based mRNA vaccine. This method is capable of distinguishing the pI of LNPs manufactured with one or more different ionizable lipids for the purpose of confirming LNP identity in a manufacturing setting. Additionally, the method is quantitative and stability‐indicating making it suitable for both process and formulation development.

**Abbreviations**
DSPC: 1,2‐distearoyl‐sn‐glycero‐3‐phosphocholine
icIEF: imaged capillary isoelectric focusing
LNP: lipid nanoparticle
mRna: messenger RNA
PEG‐lipid: polyethylene glycol‐lipid
siRNA: small interfering RNA

## 1 Introduction

Drug and vaccine development in which the active drug substance is encapsulated with a lipid nanoparticle (LNP) has gained momentum over the past decade as an efficient drug delivery system 1, 2. Several lipid‐based delivery systems have been clinically approved to deliver small drug molecules such as doxorubicin and vincristine 3. In 2018, the first LNP‐based drug containing small interfering RNA (siRNA) named Patisiran was approved for the treatment of hereditary transthyretin‐mediated amyloidosis 4. Additional LNPs are being evaluated clinically for delivery of a wide variety of nucleic acids, including siRNA, messenger RNA (mRNA), and plasmid DNA, for both therapeutic and vaccine purposes 5, 6, 7. LNPs that encapsulate nucleic acid macromolecules are generally comprised of four components: (1) an ionizable amino lipid (cationic lipid); (2) a zwitterionic phospholipid such as 1,2‐distearoyl‐sn‐glycero‐3‐phosphocholine (DSPC) or 1,2‐dipalmitoyl‐sn‐glycero‐3‐phosphocholine (DPPC); (3) a neutral lipid such as cholesterol; and (4) a polyethylene glycol‐lipid (PEG‐lipid) 8. The ionizable cationic lipid plays a principal role, for example, in siRNA transfection, by mediating cytosolic delivery of the siRNA through facilitated endosomal escape after LNP endocytosis. Neutral lipids, such as DSPC, 1,2‐dipalmitoyl‐sn‐glycero‐3‐phosphocholine, and cholesterol, are selected to modulate the fluidity and phase behavior of the LNP, whereas PEG‐lipids are utilized to improve particle circulation half‐life and systemic exposure 9.

LNPs are produced through a self‐assembly process and can be made to have a particle size ranging from 70 to 110 nm depending on the target delivery purpose 10. LNPs can have a complex structure with respect to particle size, surface charge, lipid composition, particle morphology, and surface hydrophobicity 11, 12. All of these attributes can affect the uptake of LNP and release of the RNA drug in various cell types 13. In addition to the transfection efficiency, the surface charge may be correlated with cell toxicity 14, 15. Last, a guideline from the FDA recommends the physiochemical characterization of liposomes, including a stability assessment should be completed 16. Zhang et al., have described how to characterize size and morphology of LNPs using techniques such as dynamic light scattering, cryoelectron microscopy, high performance size exclusion chromatography, and asymmetric flow field‐flow fractionation 12, 17. However, there is a lack of tools to measure the surface charge of LNPs. Currently, zeta potential is the only method routinely used and available to measure surface charge of LNPs.

Liposome protein interactions have been studied using imaged capillary isoelectric focusing (icIEF) 18 and CE has been applied to study other types of large particles such as bacteria, viruses, colloidal/nanoparticles, and polymeric particles 19, 20, 21, 22, 23, 24. However, CE has not been used to effectively measure the surface charge of LNPs. Here, we describe for the first time, using CE for characterizing LNPs that encapsulate nucleic acids using icIEF separation.

Earlier publications have described traditional gel isoelectric focusing to analyze the size of colloidal nanoparticles and gold nanoparticles 25, 26. However, these gel‐based electrophoretic techniques are labor intensive and qualitative in nature. This study describes an icIEF method to measure the pI of LNPs for the process and formulation development of an mRNA‐based vaccine. This method is capable of distinguishing the pI of LNPs manufactured with different cationic lipids, is quantitative, and is stability‐indicating.

## 2 Material and methods

### 2.1 Chemicals and reagents

All methylcellulose containing solutions, Maurice icIEF fluorescence calibration standards, system suitability standards, pI markers (5.85 and 8.40), Servalyt pH 2–9 ampholytes, and icIEF cartridges were obtained from ProteinSimple (Santa Clara, CA, USA). Pharmalyte ampholytes pH 3–10 and pH 5–8 were purchased from GE Healthcare (Uppsala, Sweden). Glycerol and sucrose were purchased from Sigma–Aldrich (St. Louis, MO, USA).

### 2.2 LNP preparation

LNPs containing mRNA were prepared in‐house as previously described 27. Briefly, LNP preparation includes (i) with, or without (empty LNP) mRNA, drug substance, (ii) a cationic lipid, (referred to here as Cationic Lipid‐1 or Cationic Lipid‐2), which is an ionizable lipid that complexes with mRNA to promote the formation of LNPs, (iii) one or more commercially available lipids, such as cholesterol, DSPC, and 1,2‐dimyristoyl‐rac‐glycero‐3‐methylpolyoxyethylene, that contribute to the overall pharmaceutical properties of LNP. A lipid stock solution was prepared by dissolving the cationic lipid, cholesterol, phospholipid, and 1,2‐dimyristoyl‐rac‐glycero‐3‐methylpolyoxyethylene in ethanol in a molar ratio of 50–58:30–38:10:1–2.

### 2.3 icIEF sample preparation

The icIEF sample preparation has been previously described 28, 29. Unless specified otherwise, the ampholytes solution was prepared by combining two parts of the ampholytes (pH 5–8) with 1 part of the ampholytes (pH 3–10). The sample was prepared by combining 70 µL of 0.5% methylcellulose, 8 µL of ampholytes solution, 16µL of glycerol (99%), 1 µL of each pI marker 5.85 and 8.40 with various volumes of LNP (0.5 to 2.5 µL) to make consistent cationic lipid concentrations, and various amounts of water to obtain a final volume of 160 µL. The samples were centrifuged at 5 000 g for 5 min before 120 µL was transferred to the 96‐well plate.

### 2.4 icIEF instrument and software

Maurice is an instrument from ProteinSimple that is similar to iCE280 and iCE3 instruments except the capillary is provided in a preassembled cartridge. The IEF separation capillary is 50 mm in length and is 100 µm ID x 200 µm OD silica coated with fluorocarbon. The catholyte consists of 0.1 M NaOH in 0.1% methylcellulose and the anolyte consists of 0.08 M phosphoric acid in 0.1% methylcellulose. All other reagents, such as system suitability standard, fluorescence calibration standard, and 0.5% methylcellulose, were prepared according to vendor recommendations. The capillary is automatically calibrated with a fluorescence standard preconditioned with a system suitability control to ensure the capillary is functioning properly. The samples were injected using the default pressure setting for 55 s and were prefocused for 1 min at 300 V/cm, followed by focusing time for 8 min at 600 V/cm. All electropherograms were detected with UV absorbance at 280 nm. All data analyses were performed using vendor software called *Compass for iCE*. The *Compass* software aligns each electropherogram using the pI markers so that the *x*‐axis is displayed as a normalized pI for each injection.

## 3 Results and discussion

### 3.1 Apparent pI measurement of LNPs

**Figure 1:**
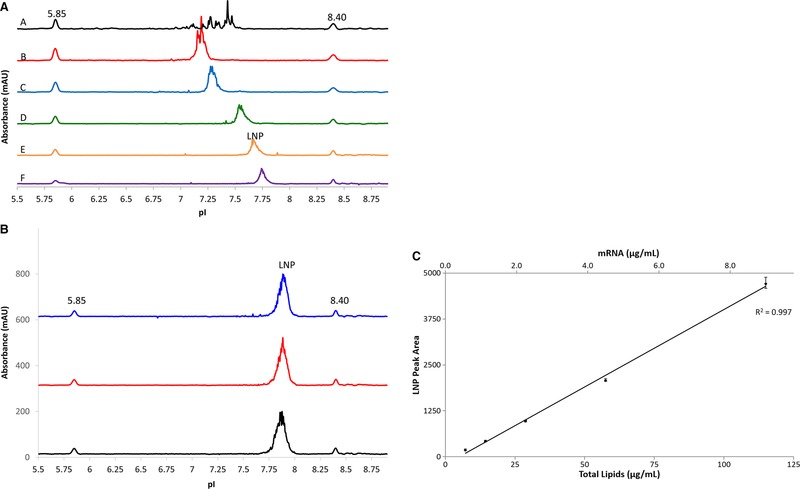
(A) Electropherograms of an LNP using various ampholytes and additives. Traces A and B show high background or precipitation and LNP containing sharp peaks using the broad range Servalyt pH 2–9 and Pharmalyte ampholytes pH 3–10, respectively. Trace C shows a focused LNP with an apparent pI of approximately 7.3 using pH 3–10 Pharmalyte ampholytes containing 10% glycerol. Trace D uses a mixture of 33.3% ampholyte pH 5–8 and 66.6% ampholytes pH 3–10 with 10% glycerol. Trace E uses a 66.6% ampholyte pH 5–8 and 33.3% ampholytes pH 3–10 with 10% glycerol. Trace F uses ampholyte pH 5–8 containing 10% glycerol. The pI of the LNP shifts to approximately 7.6–7.8 in traces D, E, and F. Two pI markers are 5.85 and 8.40. (B) Electropherogram of an LNP prepared in triplicate. An LNP sample was prepared in triplicate for the icIEF experiment. The LNP has an apparent pI of approximately 7.89 and peak shape was consistent for the three replicates. (C) Calibration curve of LNP, which ranges from 7.2–115 µg/mL of total lipids. LNP samples were diluted in icIEF ampholyte mixtures from 0.56 to 9.0 µg/mL of mRNA (equivalent to 7.2 to 115 µg/mL of total lipid). This linear range has a coefficient of determination (*R* ^2^) ≥ 0.997.

Development of an icIEF method for LNPs was initiated using broad range ampholytes to determine the apparent pI. Two different broad range ampholytes (Servalyt pH 2–9 and Pharmalyte pH 3–10) were tested and compared initially as shown in Fig. 1A (trace A and B, respectively). The Servalyt ampholytes profile showed many sharp irreproducible peaks indicating possible LNP precipitation or aggregation. The Pharmalyte mixture showed an inconsistent broad peak shape. Glycerol is known to be a stabilizing additive for protein cIEF method development 30, and was tested with the Pharmalyte mixture. The addition of 10% glycerol helped to consistently and reproducibly focus the LNP as illustrated in Fig. 1A trace C. Higher percentages (20% and 40%) of glycerol noticeably increased the viscosity and, thus, decreased the ability of the LNP to be focused in the tested separation time (data not shown). Based on the initial observation using the broad range ampholytes mixture, the estimated pI of the LNP was approximately 7.3 (Fig. 1 trace C).

The separation was further optimized by mixing different amounts of narrower range Pharmalyte ampholytes pH 5–8 into the Pharmalyte ampholytes pH 3–10. As the percentage of the narrow range ampholytes increased from 33% to 100%, the apparent pI shifted from approximately 7.6 to 7.8 (Fig. 1A trace D, E, and F, respectively). Figure 1A trace F showed the best LNP peak shape, yet the separation was more reproducible and consistent using the conditions shown in trace E. Trace E was the final ampholyte mixture used in all remaining experiments.

The ampholyte screening and optimization, shown in Fig. 1A, was performed with an LNP containing Cationic Lipid‐1, which had an apparent pI of 7.7. The LNP peak was relatively broad and not quite as homogeneous as normally observed for proteins. This broad peak shape is consistent with published gel IEF methods analyzing gold nanoparticles 25. We believe this broad, jagged peak is likely caused by the polydispersity of generating different LNP structures and sizes during the manufacturing process. It is important to note that mixing the broad range and narrow range ampholytes can introduce different pH slopes, thus, shifting the apparent pI. This phenomenon was observed in Fig. 1A traces C to F; the different ampholyte mixture showed a pI shift of approximately 0.5 units. Regardless, using the final ampholytes mixture, the pI and peak shape remained consistent between three individual preparations of a different LNP samples (Fig. 1B). Precision of the pI was evaluated with this LNP sample over 16 independent runs; the LNP had an average pI of 7.89 ± 0.028 (RSD < 0.4%).

Last, five concentrations of an LNP sample ranging from 0.56 to 9.0 µg/mL of mRNA (equivalent to 7.2 to 115 µg/mL of total lipids) were tested by icIEF and the pI was determined. Using linear regression analysis, the peak area of the standard (LNP) was plotted against the mRNA concentration (µg/mL). This linear range has a coefficient of determination (*R* ^2^) ≥ 0.997 showing strong linearity and demonstrating the ability of this technology to perform quantitative analysis (Fig. 1C).

### 3.2 Effect of lipid concentration on the pI

The pI of an LNP sample was found to vary when loading different quantities of LNP based on mRNA concentration into the ampholytes mixture; samples of higher LNP concentration display a higher apparent pI. To further investigate if the pI variation was caused by the cationic lipid or the mRNA concentration, both parameters were examined. Four different LNP batches were formulated and each batch contained a different cationic lipid to mRNA ratio (mole/mole). The four LNP batches had cationic lipid to mRNA ratios of 3.1, 6.6, 12.2, and 20.1. The LNP batches were diluted to five different cationic lipid concentrations and subjected to icIEF. The apparent pIs for all prepared LNPs were plotted against cationic lipid and mRNA concentrations.

**Figure 2:**
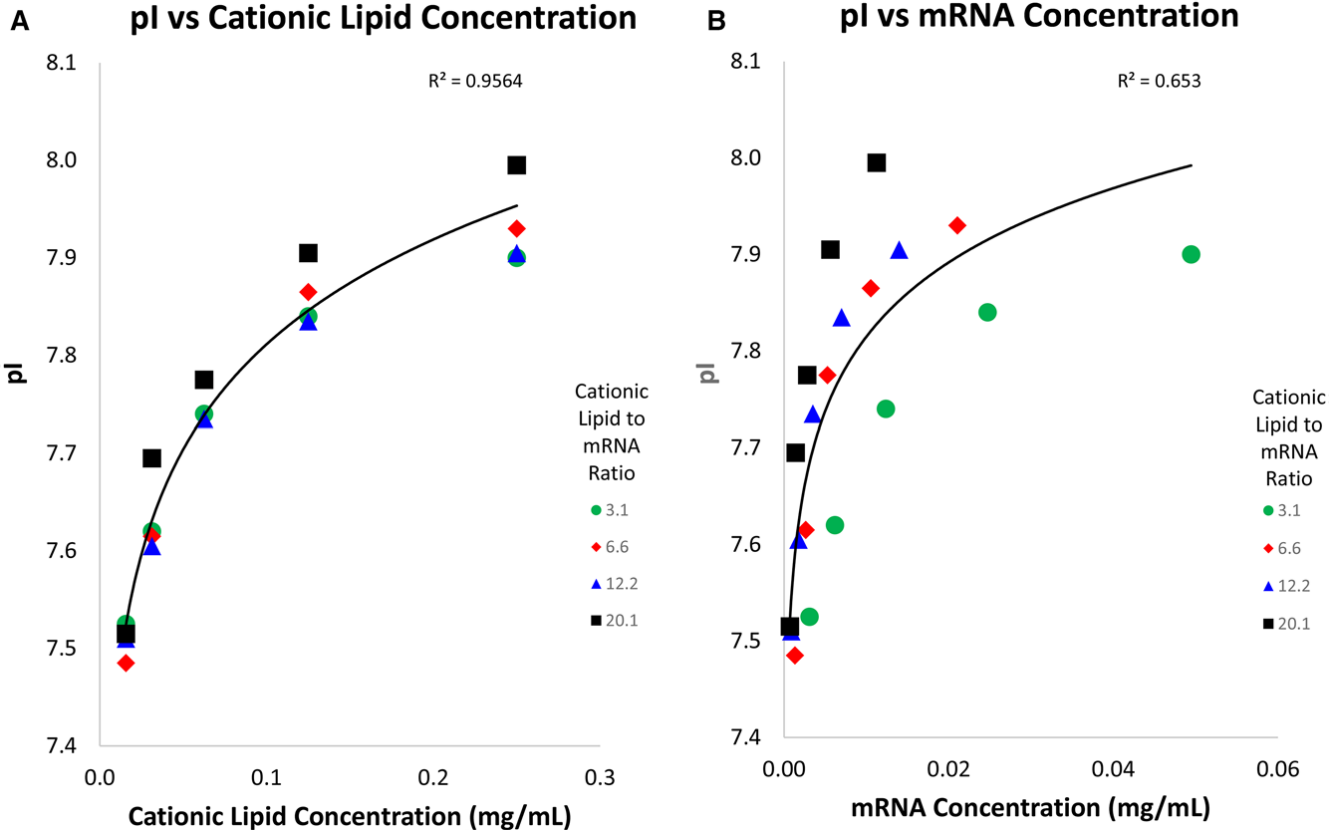
LNP pI plotted by cationic lipids and mRNA concentration: different LNP batches were formulated to contain different cationic lipid to mRNA ratios. These LNPs were then diluted to five different lipid concentrations and subjected to icIEF. The pI (*y*‐axis) was plotted against cationic lipid concentration (Fig. 2A) and the mRNA concentration (Fig. 2B). A strong correlation of pI to cationic lipid concentration was observed (*R* ^2^ = 0.956; logarithmic fit, left graph) compared to a weaker correlation of pI to mRNA concentration (*R* ^2^ = 0.653; logarithmic fit, right graph).

The apparent pI was found to have a strong correlation to cationic lipid concentration with an *R* ^2^ = 0.956, using a logarithmic fit (Fig. 2A). The apparent pI has a weaker correlation with an *R* ^2^ = 0.653, using a logarithmic fit to mRNA concentration (Fig. 2B). Results of this experiment indicate that icIEF sample loading should be normalized according to cationic lipid concentration rather than to mRNA concentration to maintain consistent pI results between different LNP batches. This data correlate well with the hypothesis that the cationic lipid is at the surface of the LNP and the mRNA is located inside of the LNP. This hypothesis is reasonable as the LNP acts as a protective hydrophobic barrier to protect the mRNA.

### 3.3 UV‐Vis spectrum of LNP with and without mRNA

**Figure 3:**
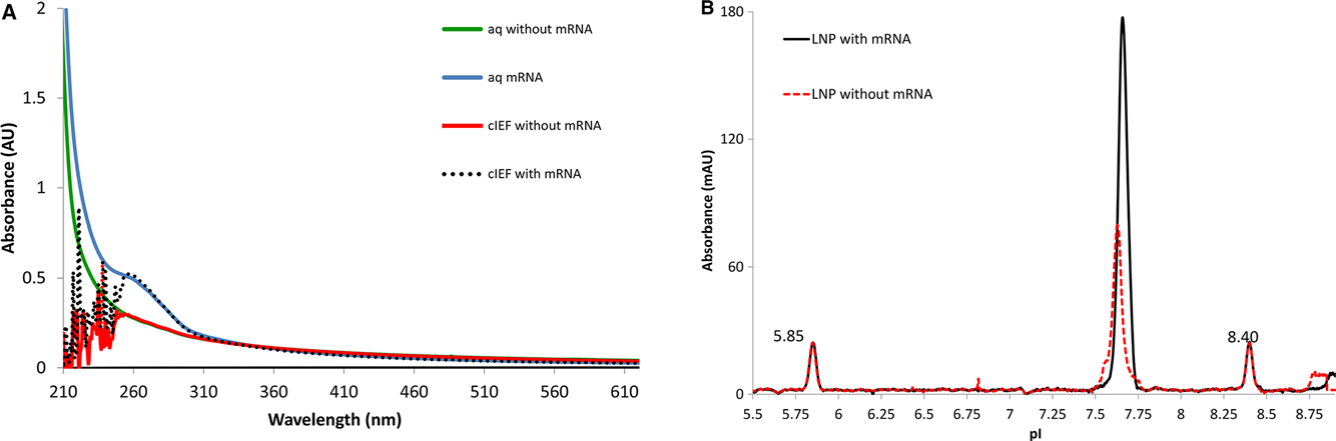
(A) UV absorbance of LNPs in both aqueous and icIEF ampholyte mixtures. LNPs containing mRNA were tested in Tris buffer (blue trace) and icIEF ampholyte mixture (black dash trace). LNPs without mRNA were tested in Tris buffer (green trace) and icIEF ampholyte mixture (red dash trace). LNPs containing mRNA show an absorbance max at 260 nm compared to LNP without mRNA, which lack a peak at 260 nm. Both aqueous and icIEF ampholyte mixtures absorbance traces were identical when comparing the wavelengths at 260 nm demonstrating that with or without mRNA, LNPs are stable and intact in the final cIEF ampholyte mixture. (B) Electropherogram of LNP formulated with and without mRNA. LNPs without mRNA (red dashed trace) have a similar pI to LNPs formulated with mRNA (black solid trace). The pI of both LNPs is approximately 7.6–7.7. Two pI markers were 5.85 and 8.40.

The icIEF instrument detects the LNP at 280 nm and the instrument does not allow this wavelength to be altered. To better understand how the LNP was being detected by the icIEF instrument, the absorbance spectrum of LNPs (containing Cationic Lipid‐1) formulated with and without mRNA was measured using an Agilent 8453 ultraviolet‐visible spectrophotometer. The LNPs were prepared in two different matrices: Tris buffer (10 mM Tris with 10% sucrose) and cIEF ampholytes matrix. The Tris buffer was measured because ampholytes used in cIEF are known to have interference at wavelengths below 280 nm. In addition, the samples prepared in Tris buffer serve as a control spectrum of intact LNPs. Figure 3A shows UV spectra for each matrix measured from 210 nm to 600 nm.

The UV spectra of LNPs containing mRNA for both Tris and ampholyte matrices showed an elevated UV absorbance from 240 to 290 nm, with an absorbance maximum at 260 nm. The absorbance at 260 nm is due to the mRNA component of the LNP. In addition, both LNPs formulated with and formulated without mRNA have significant light scatter throughout the wavelengths collected.

Comparing the UV spectra of LNPs without mRNA for both Tris and ampholyte matrices, the spectra showed only broad‐spectrum light scattering. No defined UV absorbance was observed within 240–290 nm. These data suggest that observed icIEF signal at 280 nm results from a combination of both the mRNA absorbance and light scattering of the approximately 100 nm LNP. Thus, mRNA is not needed to obtain a signal at 280 nm.

Last, LNPs measured in the ampholyte matrix displayed inconsistent signal from 210 to 256 nm. This inconsistency was expected due to interfering components in the ampholyte matrix resulting in higher background below 250 nm 30. Regardless, both the Tris buffer matrix and the ampholyte matrix absorbance traces were identical at wavelengths higher than 260 nm. These data also suggest that LNPs formulated with or without mRNA are stable and intact in the final ampholyte matrix (Fig. 3A).

The apparent pIs of LNPs formulated with or without mRNA were subsequently measured by icIEF. Figure 3B shows that LNPs with and without mRNA have similar pIs at approximately 7.7. As expected, the peak areas for LNPs containing mRNA are larger than those for LNPs without mRNA. These data further support the conclusion that the observed signal is a combination of both scattered light and mRNA absorbance.

### 3.4 Effect of cationic lipid type on LNP pI

The LNPs contain several ionizable groups that can contribute to the apparent pI: the phosphate backbone of the mRNA, the cationic lipid, the zwitterionic phospholipid, and potential degradants from the various lipids (e.g., fatty acids resulting from hydrolysis of DSPC or the PEG‐lipid). The contribution of the phosphate backbone within the mRNA is negligible as shown in Fig. 2B and 3B.

**Figure 4:**
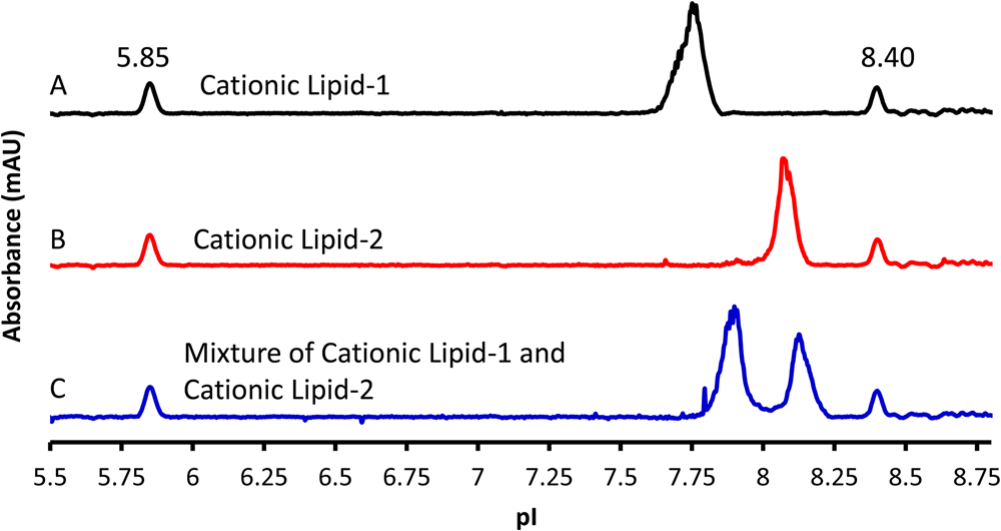
LNP with different cationic lipids have unique pIs. Trace A shows an LNP containing Cationic Lipid‐1with a lower pKa value has a pI of 7.6–7.7. (B) LNP containing Cationic Lipid‐2 with a higher pKa value has a pI of 8.1. (C) Separation of a mixture of LNP containing different cationic lipids. Two pI markers were 5.85 and 8.40.

To demonstrate the apparent pI of the LNP is dependent on the cationic lipid used in the LNP, a second LNP was evaluated containing Cationic Lipid‐2. Cationic Lipid‐2 has a pKa of approximately 0.4 units higher than that of Cationic Lipid‐1. Figure 4 trace A shows an LNP containing Cationic Lipid‐1 with a pI of approximately 7.75. The LNP containing Cationic Lipid‐2 demonstrated a higher apparent pI of approximately 8.1, as illustrated in Fig. 4 trace B, which suggests the pKa of the cationic lipid is the main contributing factor for the observed pI. When a mixture of these two LNPs was prepared and analyzed by icIEF, both LNPs were baseline resolved as shown in Fig. 4 trace C. A slight basic shift for both LNP peaks was observed; at this time, the mechanism for this observation is not known and further investigation is required. Regardless, this finding suggests the method is capable of separating LNPs by their pI based on the discrete cationic lipid pKa values (Fig. 4). The results demonstrate that the icIEF method can be used to confirm the identity of the cationic lipid present within a given LNP sample.

### 3.5 Detecting LNP stability

**Figure 5:**
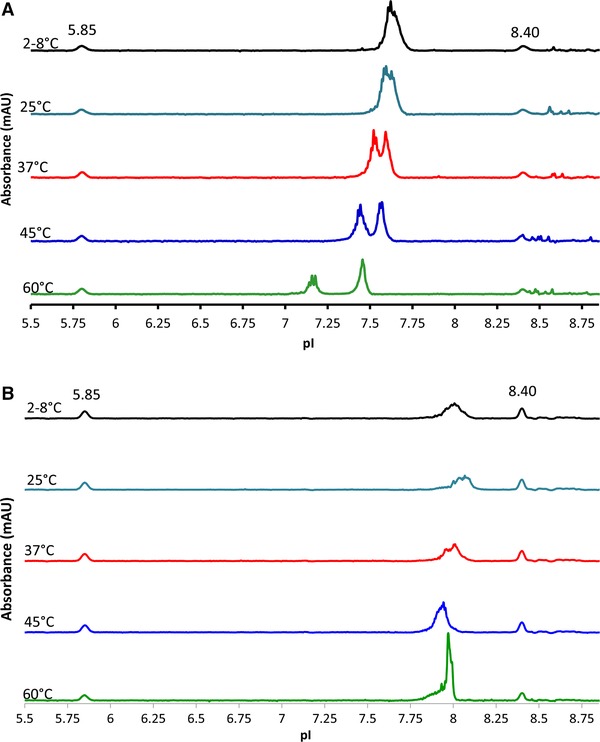
(A) Stability of LNPs containing mRNA. The LNPs containing mRNA were exposed to elevated temperatures for 24 h. The LNP stored at 2–8°C showed a symmetrical peak shape with a pI of approximately 7.7. As the temperature increased, the LNP with mRNA peaks became more acidic and split into two distinct peaks. (B) Stability of empty LNPs. LNPs without mRNA stored at 2–8°C showed a symmetrical peak shape with a pI of approximately 8.0. As the temperature increased, the LNP without mRNA showed a different degradation pattern compared to the LNPs containing mRNA. Two pI markers were 5.85 and 8.40.

The new icIEF method can detect changes in LNP stability upon heat stress as shown in Figs 5A and B. In Fig. 5A, whenan mRNA‐containing LNP sample was stressed at 37°C for 24 h, the entire LNP profile shifts to lower apparent pI values and a new peak is detected (analogous to acidic variants in the context of protein analysis). Moreover, the “acidic variants” became more acidic with increasing temperature. At 60°C, the “acidic variants” were baseline separated from the main peak with a pI of 7.4 (Fig. 5A). This suggests that the higher the stress temperature, the greater the “acidic variant.”

The LNP stability experiment described above was repeated using LNPs that were formulated without mRNA. The corresponding electropherograms shown in Fig. 5B had a different degradation pattern compared to the LNPs containing mRNA. At 45°C after 24 h, the empty LNP peak showed a slight acidic shift of approximately 0.1 pI units. A sample stressed at 60°C for 24 h showed an uncharacteristic peak profile containing a sharp spike in absorbance, which may indicate LNP destabilization or aggregation. Unlike the mRNA‐containing LNPs, these preparations did not show splitting into two peaks or generation of “acidic variants.” It is conceivable that the previously seen acidic peak could be due to the mRNA being exposed on the surface of the LNP. Future work employing LC ion exchange could be exploited to further investigate the behavior of these stressed samples.

## 4 Concluding remarks

Characterization of LNPs is challenging because they are heterogeneous mixtures of large complex particles. There are limited methods for surface charge LNP characterization that have been explored including CE. To better characterize the surface charge of LNP drug delivery systems, we have developed a new icIEF method. This method uses a commercially available cIEF instrument and can measure the pI of LNPs formulated with or without mRNA. The icIEF method can reproducibly measure the apparent pI of LNPs, provided that the cationic lipid concentration is known. With detection at 280 nm, the observed signal is proportional to the LNP concentration. Surface charge of an LNP is found to be primarily driven by the cationic lipid, implying that this lipid is at the surface of the LNP. In addition, this method is capable of differentiating LNPs containing different cationic lipids and is suitable as a test for LNP identity. More importantly, it is a stability‐indicating assay, which can be used to support process and formulation development for LNP‐based mRNA vaccines. To the best of our knowledge, this is the first reported use of icIEF applied to an LNP‐based drug delivery system.

*The authors have declared no conflict of interest*.
